# Glymphatic system, AQP4, and their implications in Alzheimer’s disease

**DOI:** 10.1186/s42466-021-00102-7

**Published:** 2021-01-19

**Authors:** Inês Silva, Jéssica Silva, Rita Ferreira, Diogo Trigo

**Affiliations:** 1 Medical Sciences Department, University of Aveiro, 3810-193 Aveiro, Portugal; 2 Neuroscience and Signalling Laboratory, Institute of Biomedicine (iBiMED), Department of Medical Sciences, University of Aveiro, 3810-193 Aveiro, Portugal

**Keywords:** Glymphatic system, Alzheimer’s disease, Aquaporin-4, Amyloid-β, Clearance

## Abstract

Lacking conventional lymphatic system, the central nervous system requires alternative clearance systems, such as the glymphatic system, which promotes clearance of interstitial solutes. Aquaporin-4 water channels (AQP4) are an integral part of this system and related to neuropathologies, such as Alzheimer’s disease (AD). The clearance of Alzheimer’s associated proteins amyloid β and tau is diminished by glymphatic system impairment, due to lack of AQP4. Even though AQP4 mislocalisation (which affects its activity) is a phenotype of AD, it remains a controversial topic, as it is still unclear if it is a phenotype-promoting factor or a consequence of this pathology. This review provides important and updated knowledge about glymphatic system, AQP4 itself, and their link with Alzheimer’s disease. Finally, AQP4 as a therapeutic target is proposed to ameliorate Alzheimer’s Disease and other neuropathologies AQP4-related.

## Background

**Fig. 1:**
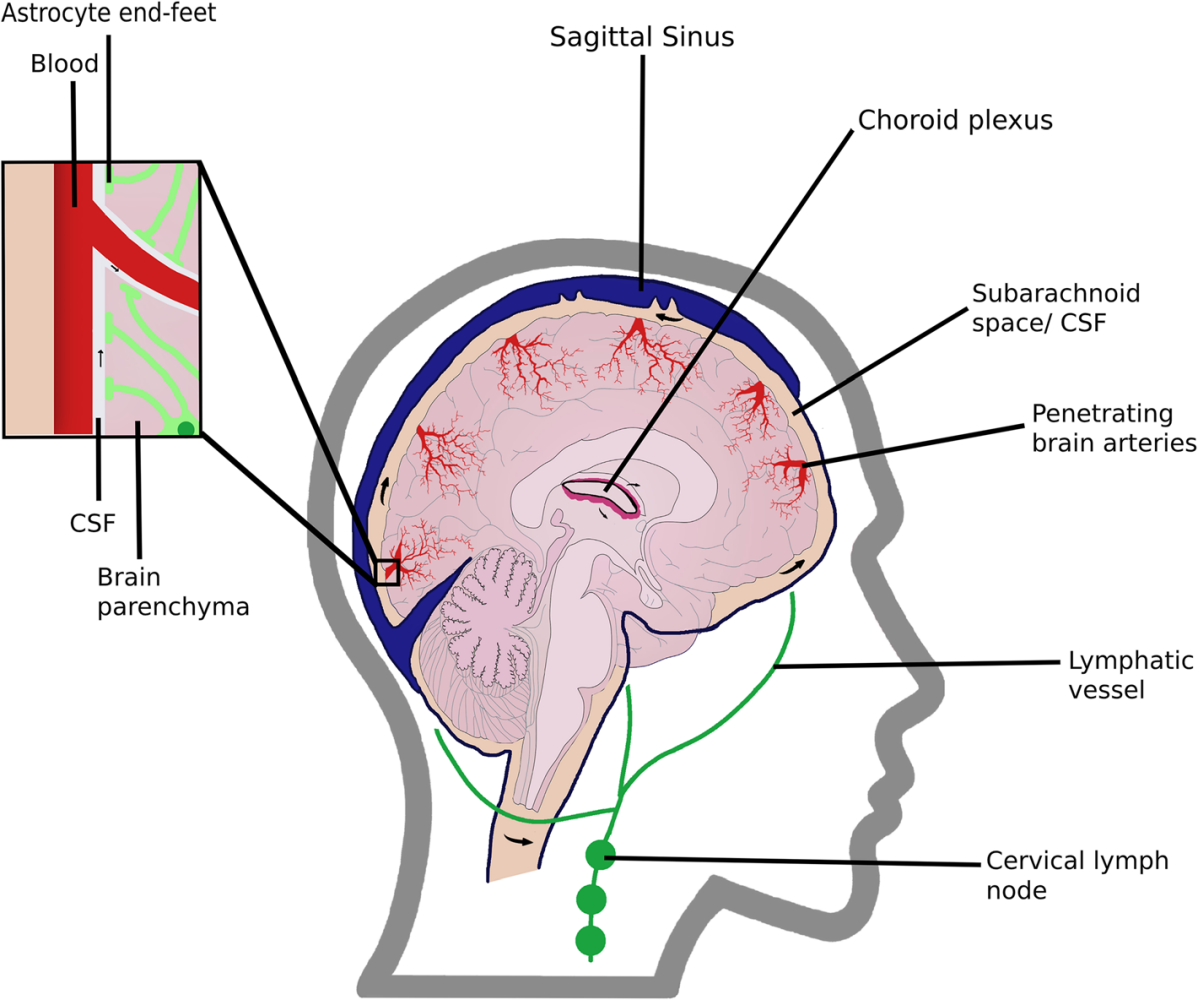
Sagittal view of the glymphatic system, showing in detail the astrocytes and their end-feet. CSF flows from the Choroid plexus into the subarachnoid space, under the sagittal sinus. From there, it flows along the outside of brain arteries into the interstitium, mixing with ISF, and clearing the solutes present in the brain parenchyma

Neural cells in the brain are supported by two forms of brain-specific extracellular fluids, the interstitial fluid (ISF) and the cerebrospinal fluid (CSF) [1]. The ISF, along with the extracellular matrix, forms the interstitial system, a connecting space between the vascular system and neural networks [2]. This fluid surrounds the brain parenchyma cells and represents 12–20% of brain water [1]. On the other hand, the CSF, produced in the choroid plexus, fills the cerebral ventricles and represents 10% of brain water (Fig. 1) [1, 2]. CSF provides mechanic protection to the brain, maintains its homeostasis, and removes waste products, being in constant communication with the ISF. Recently, its role as carrier of solutes was identified to interfere with major brain processes, such as neurogenesis [3].

Considering that unremoved waste products can promote neurodegenerative diseases, it is important to understand the mechanisms behind brain waste clearance [4]. While peripheral organs rely on the lymphatic system to clear waste products from cellular metabolism released into the ISF, the central nervous system (CNS) was believed to completely lack lymphatic vessels. Even though this appears to remain true for the brain parenchyma functional lymphatic vessels in the brain meninges have recently been described (5; 6). These vessels express all the molecular markers of the lymphatic endothelial cells of the conventional lymphatic vessels and play an important role in CSF drainage as they can successfully clear macromolecules and immune cells from the subarachnoid space and into the cervical lymph nodes [5–7]. Since these vessels do not reach the parenchyma, complementary mechanisms are needed.

The brain has other clearance systems, one of which is interstitial solute transport across the blood–brain barrier (BBB) [8], which is then drained into the blood stream. However, this route can be hindered by the large distance between interstitial solutes and the BBB; additionally, the tightly sealed endothelium of brain capillaries (which constitutes the BBB) precludes normal systemic interstitial and lymphatic flow into the brain [9]. To bypass this situation, other clearance routes are favoured, such as CSF-ISF bulk flow, known as the glymphatic system [4].

The breakdown of the CSF-ISF exchange has been associated with various neurodegenerative diseases, such as cerebrovascular disease, Lewy body disease [10], and notably Alzheimer’s disease (AD) [11]. AD is the most common type of dementia, contributing to 60–70% of all cases [12], and it is mainly characterized by amyloid-β (Aβ) and tau protein deposition [10].

Playing a pivotal role in AD, the imbalance between Aβ production and clearance results in toxic accumulation [8]. This protein is produced from amyloid precursor protein (APP), a transmembrane protein that undergoes post–translational processing [13]. In physiological conditions, APP is cleaved sequentially by α- and γ- secretases, resulting in rapidly degraded peptides; however, absence of α-secretase cleavage leads to APP internalisation into endocytic compartments, where it is alternatively cleaved by β-secretase 1 (BACE1). The resulting product is subsequently cleaved by γ-secretase, resulting in the more aggregating-prone isoforms Aβ40 and Aβ42 [8, 14].

Tau is an intracellular protein that regulates the assembly and stability of neuronal microtubules via its phosphorylation [14, 15]. In AD, tau is hyperphosphorylated, accumulating in the form of intracellular neurofibrillary tangles. This compromises its microtubule-binding ability and promotes neurodegeneration, and leads to accumulation of microtubule-transported APP, further contributing to neurodegeneration [14].

This review is focused on the glymphatic system and its importance in brain waste clearance. It addresses the role of AQP4 in this system, its suggested roles in Aβ and tau clearance, and potential as a therapeutic target for Alzheimer’s Disease, as well as other AQP4-related neuropathologies.

## Main text

### Glymphatic system

The glymphatic system is a novel structure, first described in vivo in 2012. Iliff, et al. demonstrated that subarachnoid CSF enters the brain interstitium along the outside of penetrating arteries (para-arterial influx), mixing with ISF (Fig. 1). CSF-ISF then flows through the interstitium, being drained via paravenous pathways through a yet undescribed route to the meningeal lymphatic vessels (MLV), reaching the cervical lymphatics [11, 16]. This fluid movement through the brain allows the clearance of extracellular proteins, such as Aβ and tau, from the interstitium, being particularly important in deeper areas of the brain, where the interstitial solutes cannot directly reach the BBB [4, 11].

One of the most important factors influencing CSF movement is arterial pulsatility, the movement of the vessel walls caused by the cardiac cycle. By pulsating, the arteries momentarily increase the pressure on surrounding fluid, extravasating to the paravascular space [17]. The positive pressure of CSF production itself drives its movement from the choroid plexus, supported by the presence of cilia and processes such as deep respiration [18].

Astrocytes allow the movement of fluid between paravascular spaces and the interstitium via water channels (more specifically AQP4) [11]; following these findings, the system was named “glymphatic”, after its glial dependence and functional resemblance to the peripheral lymphatic system.

In addition to solute clearance, other functions of the glymphatic system were further identified. CSF influx was observed to be a vessel for glucose and other nutrients, to be uptaken by neurons and astrocytes [19]. Additionally, apolipoprotein E, essential for synaptic plasticity and cholesterol transport, can also be carried by CSF into the brain’s interstitium [20]. Finally, the glymphatic system is hypothesised to provide rapid lipid transportation across the brain and to facilitate glial signalling [21].

#### Factors that influence the efficiency of glymphatic system

*Xie* et al. described the association of sleep with an increase in the interstitial space, lessening fluid movement resistance, resulting in increased CSF-ISF volume flowing through the interstitium, leading to a more efficient solute clearance [22]. Furthermore, sleeping position can also influence this system, as lateral and supine positions were associated with higher clearance rates, reflecting improved glymphatic system efficiency [23].

General anaesthesia was initially considered to increase CSF clearance, via the increase in the interstitial space, similar to sleep [22]; however, a more recent study refuted this notion, demonstrating that this increase is not as significant as originally believed, and that CSF circulation is less active in anesthetised mice [24]. A proposed explanation for these apparently conflicting results is the fact that both neurodegeneration and aging can change anaesthesia sensitivity, with varying outcomes depending on the severity of their conditions [24].

Impairment of MLV-associated fluid movement, using models of MLV ablation, was found to slow ISF efflux and brain perfusion by CSF, compromising overall CNS clearance [16]. This was shown to affect macromolecule clearance, while fluidic pressure and water content remained unaltered [6].

Hypertension was also shown to have a negative influence on the glymphatic system, as it is linked with artery stiffening, reducing their pulsatility and, consequently, CSF movement [25].

### The role of Aquaporin-4 (AQP4)

Characterisation of the glymphatic system elucidated the role of the water channel aquaporin-4 in this clearance network. Animals lacking astrocytic AQP4 exhibited lower CSF influx and reduction of solute clearance in the parenchymal interstitium, suggesting that glymphatic system is AQP4-dependent [11].

Aquaporins (AQP) are characterized by six transmembrane pore-forming helices [26]. The aquaporin channel family, with more than 150 types, is responsible for water diffusion, being permeable to small solutes such as glycerol and urea [27].

AQP4 is the most abundant water channel in the brain, with a molecular weight of 30 kDa and a tetrameric structure [26]. This protein functions as a selective permeable water channel, contributing to ionic and osmotic homeostasis by facilitating water diffusion through the brain [27, 28]. This process is linked to various brain functions [29, 30]: it is influenced by the production and drainage of CSF fluid and it is involved in regulating cell volume and extracellular space dimensions. Osmotic homeostasis is essential for neuronal activity [31], and modulation of water transport affects ionic concentration in the extracellular fluid, which in turn influences the diffusion of neuroactive compounds towards the brain. Additionally, in pathophysiological conditions, water transport mechanisms might be directly implicated in brain oedema, a common feature of several neurological conditions such as head trauma, stroke, and brain cancer [29]. Taking everything into account, water homeostasis is a crucial mechanism for neuronal activity and function, and there so, it is important to study the distribution and regulation of water channels, such as AQP4, in the brain.

Moreover, there is strong evidence indicating that AQP4 is important for neuroexcitation, astrocyte migration, synaptic plasticity, and memory/learning performance [32]. Furthermore, AQP4 induces expression of inflammatory genes in injured murine brain, underexpressed in AQP4-deficient mice, suggesting a key role for AQP4 in neuroinflammation [33]. This notion is strengthened by the fact that AQP4 is associated with decreased IL-1β, IL-6 and TNF-alpha levels [34].

**Fig. 2:**
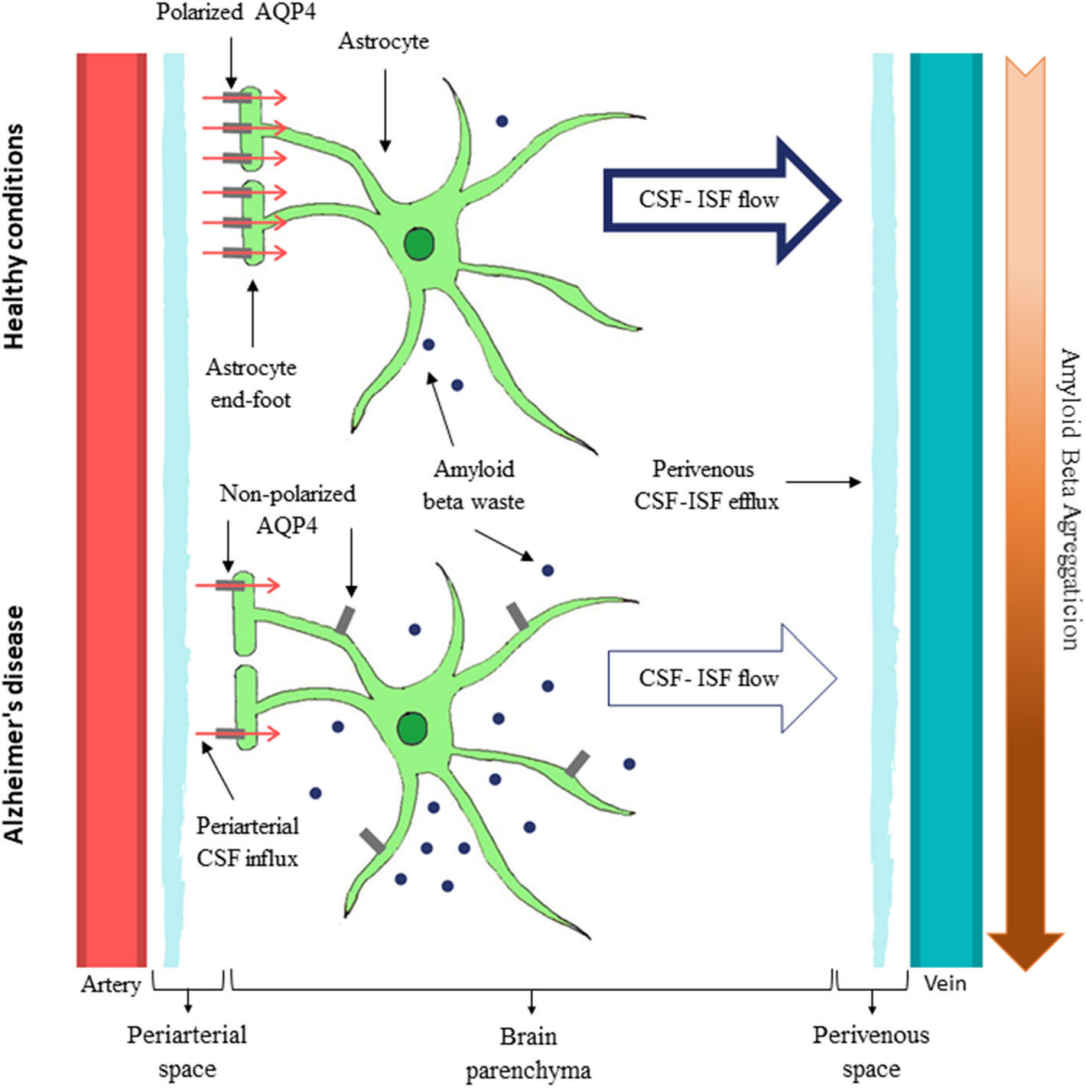
Glymphatic System in Healthy vs AD condition. In healthy condition, CSF flows across highly polarized AQP4 molecules at astrocyte end-feet, mixing with ISF, which allows the clearance of Aβ. However, in pathological situations, such as in AD, there is a loss of AQP4 polarization, resulting in restricted CSF-ISF flow, contributing to Aβ accumulation

The majority of AQP4 is found on astrocytic end-feet (Fig. 2), which is known as polarized distribution of AQP4 [35]. This localization allows AQP4 to be in contact with the perivascular space adjacent to the blood vessels, facilitating CSF influx into the brain parenchyma and its efflux back to the perivascular space [11]. Due to its particular localization, AQP4 connects astrocyte cytoplasm with the ISF, allowing a dynamic fluid distinct from the systemic tissue water dynamics, which facilitates interstitial movement, essential for the glymphatic flow [9].

In spite of the heterogeneous characteristics of astrocytes, AQP4 is expressed across all astrocytes (being one of the few markers to do so) [36], which highlights its importance in brain homeostasis, strengthening the theory that the glymphatic system is a general brain clearance pathway.

#### AQP4 and its implications for neuropathologies

Interest in AQP4 has emerged over recent years, with several reports associating this water channel with various pathologies, particularly in the nervous system [28, 37–39]. Most of these studies involved AQP4 deficient mice, observations of post-mortem brain tissue, or in vitro studies. As described before, physiological AQP4 is characteristically polarized; however, under neuropathological conditions, expression and localisation of AQP4 are altered [11, 28].

Hydrocephalus has been linked to changes in AQP4 expression, with AQP4 being upregulated and reabsorbing some of the excessive fluid as a coping mechanism [40], but it remains unclear whether AQP4 accelerates or diminishes this pathology [28]. Experiments with knockout mice showed an accelerated progression of this pathology [37], while, on the contrary, AQP4 upregulation has been suggested to contribute to early hydrocephalus development [28]. Modulation of AQP4 has been proposed as a possible therapy, increasing CSF clearance, in advanced stages, or decreasing water movement in the areas of CSF production at disease onset [28, 37]. Compounds modulating AQP4 function are under development for hydrocephalus disease but have yet to enter clinical trials [37].

AQP4 has also been implicated in neuromyelitis optica, an autoimmune disorder where it is the target antigen [28]. AQP4 blocking antibodies are in preclinical development [28], and the complement inhibitor eculizumab is undergoing clinical trials.

Another relevant pathology is ischemic stroke, where AQP4 is overexpressed at site of infarction [28]; AQP4 blockage could be a new therapeutic strategy to treat this condition. Of the available treatments for this pathology, none deals with the acute complication of oedema. TGN-020, an AQP4 inhibitor, has been explored in this context in an ischemic rat stroke model [41].

To summarise, alterations in AQP4 expression are implicated in several pathologies, making this water channel an interesting potential pharmacological target. Although some potential AQP modulators have been described, there are many limitations in this complex as a therapeutic target due to its low druggability [42].

### Glymphatic system and Alzheimer’s disease

As described above, the accumulation of Aβ and tau protein are major pathological processes in AD physiopathology. In this section, the current knowledge linking these processes and the glymphatic clearance will be explored, focusing on the role of AQP4.

#### The role of AQP4 in Aβ aggregation

*Illif* et al. hypothesized that Aβ could be cleared from the brain by the glymphatic system through an AQP4-dependent ISF bulk flow [11]. This hypothesis was sustained by the observation that Aβ clearance was halved in AQP4-knockout mice, suggesting that a significant proportion of Aβ is removed by the glymphatic system. Furthermore, the lack of AQP4 in APP/PS1 mice, a model of AD, aggravates the phenotype, with increased Aβ aggregation, loss of synaptic proteins, and, consequently, aggravation of cognitive deficits [11].

AQP4 depolarisation also occurs in AD, becoming mislocated on astrocyte parenchymal processes [43, 44]. Various studies have associated AQP4 depolarisation with AD pathology [43, 45, 46], notably in human post-mortem frontal cortex tissue [45]. The same study has suggested that AQP4 overexpression on astrocytic parenchymal processes is a feature of the ageing brain, and that preserved AQP4 perivascular localisation in elder individuals (over 85 years old) could be associated with increased cognitive performance [45]. Furthermore, this enhanced expression of parenchymal AQP4 was suggested to function as a compensating mechanism, due to astrocyte age-related alterations, common in mice [47] and humans [45]. However, this hypothesis remains speculative, with more studies being required to confirm it.

Depolarisation of AQP4 was thought to affect the clearance efficiency of the glymphatic system, leading to restricted CSF flow and increased accumulation of waste products (see Fig. 2) [11, 28]. Therefore, some studies have suggested that loss of AQP4 polarisation is a factor for reduced Aβ clearance, increasing the ageing brain vulnerability to Aβ aggregation [11, 45]. However, this hypothesis is not consensual, with other studies proposing an alternative role for AQP4 in AD: AQP4 depolarisation is instead driven by the formation of Aβ insoluble aggregates [43, 46, 48], which promotes structural astrocytic rearrangements [46].

As such, the nature of the relationship between AQP4 and AD remains controversial, since it is still unclear whether the loss of polarisation is a consequence or a cause of Aβ accumulation [43]. These findings may imply a non-linear, possibly cyclical, pathway, where the initial amyloid aggregation is amplified by impaired glymphatic function, such as loss of AQP4 polarisation, as well as promoting even more glymphatic impairment. Further studies in this area are necessary to clarify this mechanism, namely the role of AQP4 in the glymphatic system.

Moreover, disruption of MLV in mouse models of AD promoted meningeal Aβ aggregation and increased hippocampal Aβ plaque load, suggesting an aggravating role in AD pathology. However, this mechanism is seemingly independent from astrocytic AQP4, as its localization and number remained unaltered [16].

#### AQP4 and TAU protein

Not as deeply described as Aβ clearance, Tau is believed to be mainly cleared via intracellular degradation by proteasomes or lysosomes. It can also be released into the interstitium, during excitatory activity or AD-related neuronal death, following which it can be cleared through extracellular mechanisms [8]. The glymphatic system has been demonstrated to be responsible for clearing interstitial tau, in a study using AQP4 deficient mice, which found higher levels of tau after traumatic brain injury (a risk factor for neurodegenerative diseases) [15]. This finding was corroborated by a posterior study, showing that AQP4 deletion leads to Tau protein accumulation [49].

Moreover, AQP4 is anchored to astrocytic end-feet by the dystrophin-associated complex (DAC), which contains dystroglycan (DAG1) and alpha-syntrophin (SNTA1). Elevated levels of SNTA1 and DAG1 are associated with increased tau in the temporal cortex [50], and it has been proposed that the MLC1 gene (whose expression has also been associated with increased levels of tau) encodes an astroglial membrane transporter linked to AQP4 and DAC. These results suggest that AQP4 is associated with tau pathology, not only on its own, but also via its interacting proteins and genes. However, further studies are needed to clarify these associations [49].

#### The connection between sleep, age, the glymphatic system and AD

Sleep has been identified as an important factor for glymphatic activity, associated with increased CSF influx and improved interstitial waste clearance (including soluble Aβ) [51].

Studies in both human and animal models have found increased Aβ levels after sleep deprivation [52, 53], and recent findings described that some genetic variations of AQP4 can directly modify sleep quality and influence the clearance of Aβ [50]. However, sleep-deprived mice have also been shown to suffer AQP4 depolarisation [54], indicating that sleep, in turn, influences AQP4.

Additionally, age is the biggest risk factor for aggregation-associated neuropathologies, such as AD [55], and age-related changes in glymphatic function are important contributors, due to reduced waste clearance [44, 56]. In the ageing mouse brain, CSF-ISF exchange suffers a wide decline, with a 40% decrease in the clearance of intraparenchymally injected Aβ [44]. This decline was associated with reduced CSF production and arterial pulsatility, which can affect glymphatic influx, and ultimately contribute to AD [44, 56]. The reported CSF-ISF exchange decline in older mice was also accompanied by loss of perivascular AQP4, indicating that AQP4 polarisation is impaired in the aged brain [44]. As discussed above, AQP4 depolarisation is associated with glymphatic impairment, but the exact role of this protein in AD pathology should be further investigated.

#### AQP4, GLT-1 and AD

Most excitatory mammalian brain neurotransmission is mediated by glutamate amino acids. Maintenance of physiological levels of glutamate is crucial to prevent excitotoxicity, which occurs when there is excessive release of glutamate, and is believed to be implicated in AD [57, 58]. Specifically, at both gene and protein levels in hippocampus and gyrus frontalis medialis, expression of astrocytic glutamate receptors (GLTs) is altered in early-stage AD patients [57]. Additionally, uncontrolled glutamate levels can disrupt BBB cohesion [59, 60] and perpetuate a positive feedback of Aβ-induced neuronal hyperactivation in the brain [61]. One of the few drugs currently available for treatment of moderate to severe AD progression is memantine, which blocks the glutamate-receptor NMDAR [58–64].

AQP4 has been observed to co-express with GLTs in astrocytes [35], forming a complex [65]. Posteriorly, down-regulation of glutamate uptake and GLT-1 astrocytic expression was observed in AQP4 −/− mice, suggesting a significant interplay between AQP4 and GLT-1 [66]. This correlation was verified when reduction of GLT-1 expression was accompanied by AQP4 down-regulation, following exposure to AQP4-specific autoantibodies, in the human cell line HEK-293 [67].

Finally, a study on AQP4 ^−/−^ mice concluded that loss of long-term potentiation and memory formation was mediated by the decrease of GLT-1 levels, and, consequently, glutamate clearance, since these functions were rescued by the GLT-1 stimulator, ceftriaxone; being so, this study concludes that AQP4 can modulate synaptic plasticity and memory, via GLT-1 [68].

In summary, literature suggests that disruption of the AQP4/GLT-1 association has implications in AD, with a strong influence on cognitive performance. This functional complex is especially important for glutamate/water homoeostasis and, as such, for healthy brain function. GLT-1 and AQP4 in astrocytes might be neuroprotective in the progression of AD, dealing with excessive extracellular glutamate, but more studies are necessary to ascertain this hypothesis.

## Conclusions

Due to the important role of AQP4 in the glymphatic system and its implications for AD, future research should take this water channel into consideration. We propose AQP4 as an interesting therapeutic target for Alzheimer’s disease, due to its plausible effects in Aβ and tau clearance and ameliorating of neuronal function, making it a target of enormous relevance to the field of ageing and neurodegeneration. A possible future therapeutic target might be found by modulating AQP4, physiologically upregulated in the ageing brain and mislocated in AD [45, 69].

The BBB is highly selective [69], preventing the crossing of drugs from the circulating blood into the brain extracellular fluids, and even though some pathologies are associated with impaired BBB, which facilitates drug delivery, this is not the case for early-stage AD [70]. Anti-amyloid antibodies, such as bapineuzumab, are good amyloid disaggregation agents, but have failed in clinical trials, as they could not physically access amyloid plaques [71]. Being so, for any drug to efficiently target AQP4 and be relevant for AD therapy, it must be capable of crossing an intact BBB.

A study published earlier this year has demonstrated chronic administration of 5-Caffeoylquinic Acid (5-CQA) to be effective in reducing Aβ deposition in the APP/PS2 AD mouse model, improving cognitive deficits and neuronal functions: 5-CQA normalised AQP4 perivascular mislocalisation and increased Aβ clearance along the glymphatic system [72], reiterating the importance of AQP4 in this clearance system.

Despite the promising progress made in the research of glymphatic system and AQP4, further translational studies are needed to explore their alterations during AD pathology [73], and the glymphatic system in the human brain needs to be characterised in more detail, in order to develop new diagnostic and therapeutic tools.

## Data Availability

N/A

## Abbreviations

5-CQA: 5-Caffeoylquinic Acid
SNTA1: Alpha-syntrophin
AD: Alzheimer’s disease
APP: Amyloid precursor protein
Aβ: Amyloid-β
AQP4: Aquaporin-4
BBB: Blood–brain barrier
CNS: Central nervous system
CSF: Cerebrospinal fluid
DTNA: Dystrobrevin
DAG1: Dystroglycan
DMD: Dystrophin
DAC: Dystrophin-associated complex
GLTs: Glutamate receptors
ISF: Interstitial fluid
LRP1: Low density lipoprotein receptor-related protein 1
MLV: Meningeal lymphatic vessels
